# Supplementary material for: Adult and Larval Tracheal Systems Exhibit Different Molecular Architectures in Drosophila
Source: Int J Mol Sci. 2023 Mar 15;24(6):5628. doi: 10.3390/ijms24065628 (PMC10052349; doi:10.3390/ijms24065628)
Supplement: Supplementary file 1 [file ijms-24-05628-s001.zip › Supplemental material Bossen et al.pdf]

Supplemental material

for

Adult and larval tracheal systems exhibit different molecular architectures in *Drosophila*

by

Judith Bossen, Ruben Prange, Jan-Philip Kühle, Sven Künzel, Xiao Niu, Jörg U Hammel, Laura Krieger, Mirjam Knop, Birte Ehrhardt, Karin Uliczka, Susanne Krauss-Etschmann, Thomas Roeder

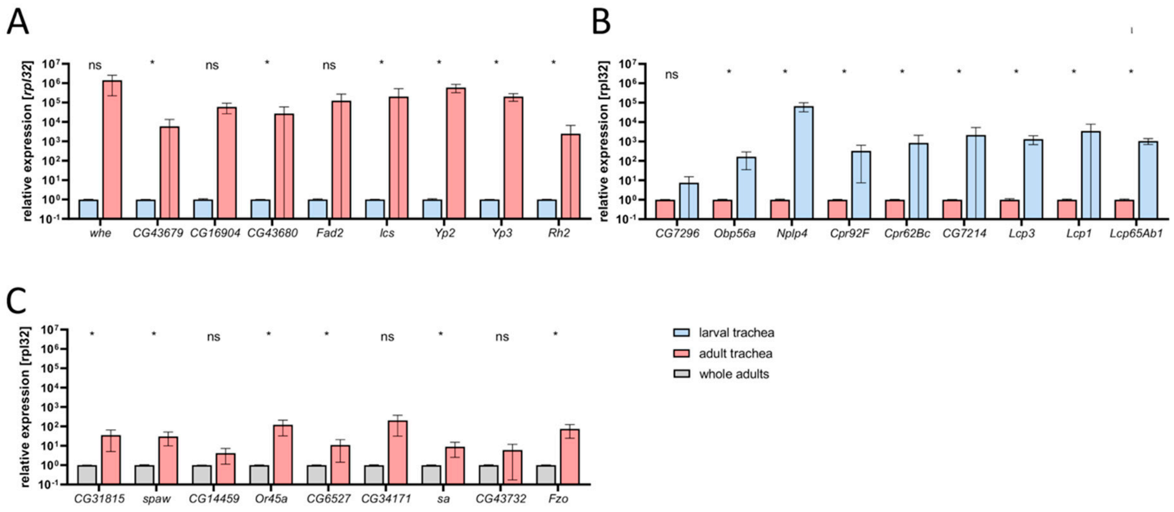

Figure S1: Validation of selected genes by qRT-PCR in larval and adult trachea.

Relative expression was measured by qRT-PCR, performed with amplified cDNA from the larval trachea (blue), adult trachea (red), and whole adult animals (grey). (**A**) Selected genes enriched in the adult trachea compared to the larval trachea. (**B**) Selected genes enriched in larval trachea compared to the adult trachea. (**C**) Selected genes enriched in adult trachea

compared to whole adult animals. Significance was evaluated by the Mann-Whitney test, * = $p < 0.05$, ns = not significant. Bars show mean and SD. n = 4.

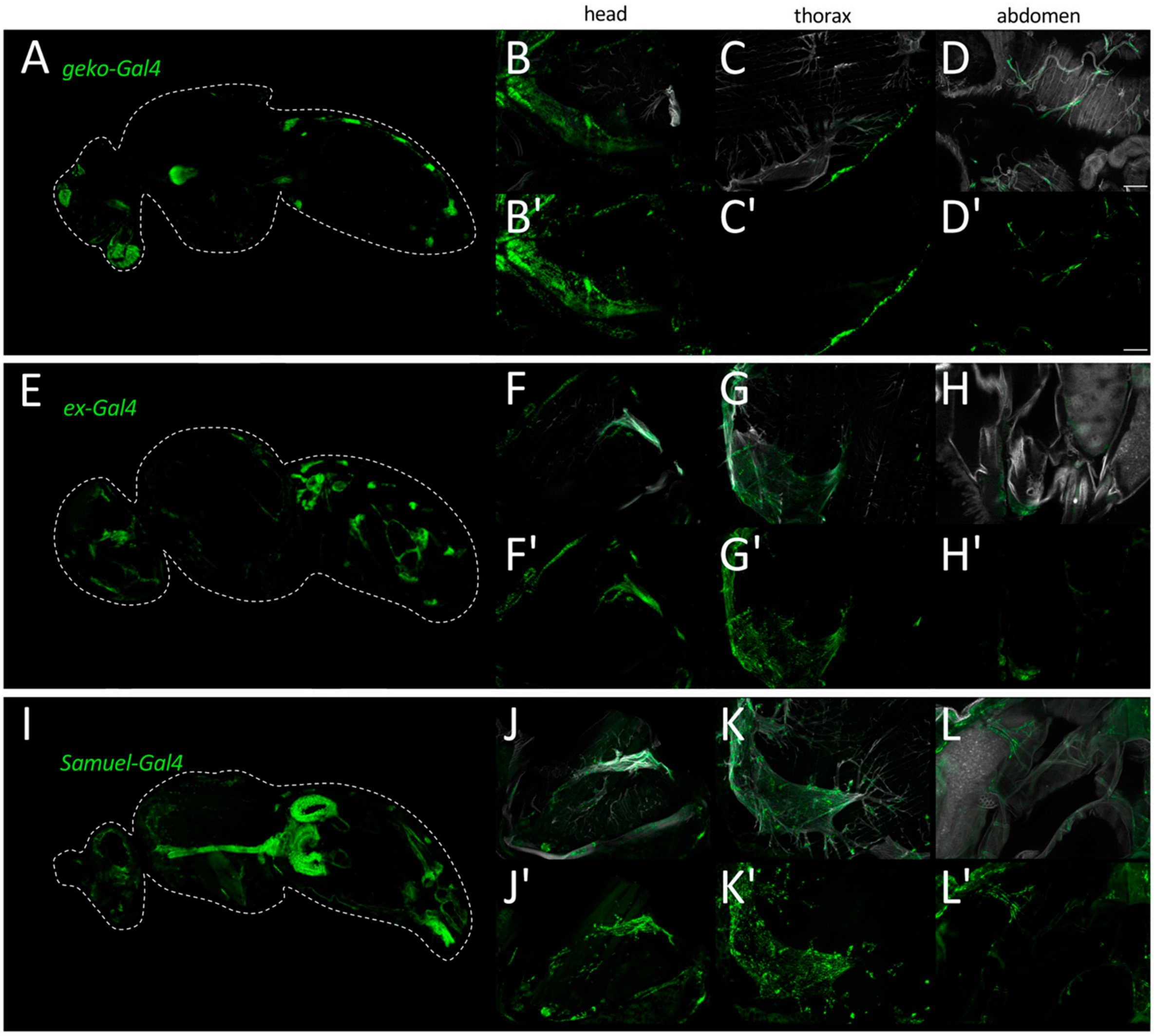

Figure S2: Expression of tracheal specific Gal4 driver lines in head, thorax and abdomen.

(**A**-**L'**) Expression of tracheal specific Gal4 driver lines in whole fly, head, thorax and abdomen. Sagittal sections of *geko-Gal4* (**A**-**D'**), *ex-Gal4* (**E**-**H'**), and *Samuel-Gal4* (**I**-**L'**) crossed to *UAS-GFP* were stained with an anti-GFP nanobody. Tracheal air sacs and branches were simultaneously visualized with UV light (white; **B**-**D**, **F**-**H**, **J**-**L**). Detailed magnification of the

head (**B**,**B’**,**F**,**F’**,**J**,**J’**), thorax (**C**,**C’**,**G**,**G’**,**K**,**K’**), and abdomen (**D**,**D’**,**H**,**H’**,**L**,**L’**) are shown. Scale = 50 µm.

Supplemental Table S1

Comparison of the transcripts of adult trachea with transcripts from total adult flies.

Supplemental Table S2

Comparison of the transcript levels of adult trachea vs. 3rd instar trachea.
